# Hydroxygenkwanin Inhibits Class I HDAC Expression and Synergistically Enhances the Antitumor Activity of Sorafenib in Liver Cancer Cells

**DOI:** 10.3389/fonc.2020.00216

**Published:** 2020-02-25

**Authors:** Chi-Yuan Chen, Chin-Chuan Chen, Wen-Yu Chuang, Yann-Lii Leu, Shir-Hwa Ueng, Chuen Hsueh, Chau-Ting Yeh, Tong-Hong Wang

**Affiliations:** ^1^Tissue Bank, Chang Gung Memorial Hospital, Taoyuan, Taiwan; ^2^Research Center for Chinese Herbal Medicine, Graduate Institute of Health Industry Technology and Research Center for Food and Cosmetic Safety, College of Human Ecology, Chang Gung University of Science and Technology, Taoyuan, Taiwan; ^3^Graduate Institute of Natural Products, Chang Gung University, Taoyuan, Taiwan; ^4^Department of Anatomic Pathology, Chang Gung Memorial Hospital, Chang Gung University School of Medicine, Taoyuan, Taiwan; ^5^Chinese Herbal Medicine Research Team, Healthy Aging Research Center, Chang Gung University, Taoyuan, Taiwan; ^6^Center for Traditional Chinese Medicine, Chang Gung Memorial Hospital, Taoyuan, Taiwan; ^7^Department of Hepato-Gastroenterology, Liver Research Center, Chang Gung Memorial Hospital, Taoyuan, Taiwan

**Keywords:** hydroxygenkwanin (HGK), liver cancer, histone deacetylase (HDAC), p21, sorafenib

## Abstract

Abnormal histone deacetylase (HDAC) expression is closely related to cancer development and progression. Many HDAC inhibitors have been widely used in cancer treatment; however, severe side effects often limit their clinical application. In this study, we attempted to identify natural compounds with HDAC inhibitory activity and low physiological toxicity and explored their feasibility and mechanisms of action in liver cancer treatment. A yeast screening system was used to identify natural compounds with HDAC inhibitory activity. Further, western blotting was used to verify inhibitory effects on HDAC in human liver cancer cell lines. Cell functional analysis was used to explore the effects and mechanisms and the *in vitro* results were verified in BALB/c nude mice. We found that hydroxygenkwanin (HGK), an extract from Daphne genkwa, inhibited class I HDAC expression, and thereby induced expression of tumor suppressor p21 and promoted acetylation and activation of p53 and p65. This resulted in the inhibition of growth, migration, and invasion of liver cancer cells and promoted cell apoptosis. Animal models revealed that HGK inhibited tumor growth in a synergistic manner with sorafenib. HGK inhibited class I HDAC expression and had low physiological toxicity. It has great potential as an adjuvant for liver cancer treatment and may be used in combination with anticancer drugs like sorafenib to improve therapeutic efficacy.

## Introduction

Liver cancer is the second most common cause of cancer-related deaths worldwide (1). Due to primary drug resistance and the propensity for metastasis, liver cancer is one of the most refractory cancers (2). Currently, surgery is the main treatment option, but for patients with advanced liver cancer that has metastasized, chemotherapy or radiotherapy is the main treatment approach (3, 4). However, most chemotherapeutic agents have strong side effects, which negatively impact patient quality of life. Therefore, the development of effective liver cancer treatments with low side effects is urgently needed.

Targeted therapy is a new approach in cancer treatment. Since it targets and deactivates specific proteins that cause carcinogenesis, targeted therapy has the advantages of high specificity and low adverse effects and is widely used in the treatment of various cancers (5, 6). Currently, the most commonly used drugs for liver cancer treatment are sorafenib and bevacizumab (7). Sorafenib inhibits the activation of multiple kinases related to tumor growth and angiogenesis and is currently the most effective drug for targeted therapy of liver cancer. In contrast, bevacizumab is a monoclonal antibody that targets vascular endothelial growth factor and inhibits tumor growth by inhibiting angiogenesis inside the tumor (8, 9). The causes of liver cancer are complex, often involving mutations in more than two carcinogenic pathways. This complexity, coupled with the heterogeneous nature of liver cancer tumors, results in the efficacy of treatments usually being limited (10, 11). Even sorafenib can only prolong survival by ~3 months (4, 12). Therefore, the development of drugs with multiple targets to improve the efficacy against liver cancer and low side effects is crucial for liver cancer research.

Histone deacetylase (HDAC) regulates the de-acetylation of histone and non-histone proteins, thereby regulating gene expression or protein stability and activity (13, 14). De-acetylation of histone proteins results in increased binding of the coiled chromonema, thereby inhibiting gene expression. Non-histone protein de-acetylation is closely related to their activity and affects the binding ability of these proteins to DNA and other proteins, indirectly regulating the activation or expression of other proteins (15, 16). Currently, 18 human HDACs have been identified, which are divided into four types, including class I (HDAC1, HDAC2, HDAC3, HDAC8), class IIa (HDAC4, HDAC5, HDAC7, HDAC9), class IIb (HDAC6, HDAC10), class III Sir2-like enzymes (consisting of seven sirtuins), and class IV (HDAC11). Among these, class I HDACs are over-expressed in most cancers, including liver cancer, making these a treatment target for many cancers (17–19). Currently, HDAC inhibitors such as trichostatin A, vorinostat (suberoylanilide hydroxamic acid, SAHA), trapoxin A, and valproic acid (VPA) have been widely used with good results in the treatment of lung cancer, breast cancer, and esophageal cancer (20–22). However, application of these HDAC inhibitors alone fails to achieve satisfactory results in the treatment of liver cancer, likely due to tumor heterogeneity. Recent studies have found that HDAC inhibitors have considerably improved efficacy against liver cancer if combined with sorafenib (23, 24). However, since most HDAC inhibitors cause strong physiological side effects, it remains necessary to carefully assess the physiological conditions of patients prior to administration.

Numerous studies have confirmed that some natural compounds have specific anticancer effects. Compared with drugs of Western medicine, these natural compounds have low physiological side effects and are thus suitable for use as therapeutic adjuvants in combination with other drugs to improve efficacy (25, 26). Currently, drugs such as artemisinin and curcumin have been used in the treatment of liver cancer and have been confirmed to considerably inhibit tumor growth and metastasis and to prolong patient survival (27–29). Certain Chinese herbal extracts such as sulforaphane have inhibitory activity against HDAC, giving them the potential for use in the treatment of a variety of diseases, including cancer (30–32). In the current study, we used a yeast analysis platform and identified hydroxygenkwanin (HGK) as an herbal extract with class I HDAC inhibitory activity. We found that HGK, a bioactive substance extracted from Daphne genkwa Sieb. et Zucc., inhibited expression of class I HDAC, induced expression of tumor suppressor p21, and promoted acetylation of p53 and p65, thereby inhibiting the proliferation, migration, and invasion of liver cancer cells. In addition, HGK had a synergistic effect with sorafenib, suggesting that its combination with sorafenib may enhance the inhibitory effects on liver cancer cells in clinical practice.

## Materials and Methods

### Cell Lines and Yeast

The hepatocellular carcinoma cell lines Huh7 and HepG2 were purchased from the American Type Culture Collection (Manassas, VA, USA). Human skin fibroblasts (HFB) were kindly provided by Dr. P. C. Yang of Taiwan University. The cells were routinely maintained in Dulbecco's Modified Eagle Medium (DMEM; GIBCO, Gaithersburg, MD, USA) supplemented with 10% fetal bovine serum at 37°C with 5% CO_2_ in a humidified incubator. Yeast cultures were grown for 12 to 14 h in yeast extract-peptone (YEP) media containing lactic acid.

### Antibodies and Drugs

Antibodies against HDAC1, HDAC2, HDAC3, HDAC8, p21, p53, p65, acetyl-p53, acetyl-p65, and β-actin, and antibodies for histone H3 and acetyl-histone H3 were purchased from Cell Signaling Technology (Beverly, MA, USA) and Genetex (Irvine, CA, USA). Antibody for cyclin D1 was purchased from ABclonal Technology (Woburn, MA, USA) and antibody for CDK4 was purchased from Proteintech (Rosemont, IL, USA). Secondary antibodies were purchased from Santa Cruz Biotechnology (Santa Cruz, CA, USA). Prestained protein marker and TOOLSmart RNA extractor were purchased from BIOTOOLS (New Taipei City, Taiwan). HGK powder (purity >98% as measured by HPLC) was purchased from Shanghai BS Bio-Tech Co., Ltd (Shanghai, China). SAHA (purity >98% as measured by HPLC) and Sorafenib was purchased from Sigma-Aldrich (St. Louis, MO, USA).

### Real-Time RT-PCR Analysis

Total RNA from Huh7 and HepG2 cells under different treatment conditions was extracted using an RNeasy Mini Kit (QIAGEN, Gaithersburg, MD, USA) according to the manufacturer's instructions. Complementary DNA (cDNA) was synthesized from the total RNA using a Reverse Transcription Kit (QIAGEN) and ToolScript MMLV RT Kit (BIOTOOLS CO., LTD., Taiwan), and used as template in quantitative real-time PCR assays using the TaqMan Gene Expression Kit (Applied Biosystems, Foster City, CA, USA) and an ABI StepOnePlus™ System (Applied Biosystems) to detect p21, p53, and p65 expression. GAPDH was used as an internal control.

### Western Blotting Analysis

Huh7 and HepG2 cells were treated with various concentrations HGK, sorafenib, or DMSO for 48 h followed by being washed twice with phosphate-buffered saline (PBS) and lysed in RIPA lysis buffer (BIOTOOLS CO., LTD., Taiwan) containing protease inhibitors. Protein samples (30 μg) were separated using SDS-polyacrylamide gel electrophoresis (PAGE), transferred to nitrocellulose membranes, and analyzed by western blot. β-actin served as a loading control. Immuno-reactive bands were detected using an ECL Chemiluminescence Kit (NEN Life Science Products, Boston, MA, USA) and developed using X-ray film. The relative intensities of the protein bands were quantified using ImageQuant 5.2 software (GE Healthcare, Piscataway, NJ, USA).

### Cell Proliferation Assay

Cell proliferation capacity was monitored using an xCELLigence Real-Time Cell Analyzer (Roche Life Science, Indiana, USA) according to the manufacturer's instructions.

### Cell Migration and Invasion Assay

Cell migration and invasion activity was analyzed using a Transwell assay as previously described (33, 34). For the migration assay, cells at a concentration of 5 × 10^4^ were resuspended in 100 μl serum-free culture medium (DMEM) with/without HGK and placed in the upper chambers. The lower chambers were filled with 500 μl DMEM medium containing 10% FBS. Twenty-four hours after treatment, the cells were fixed with methanol, and the cells on the upper surface of the membrane were removed using cotton swabs. The membrane was washed twice with PBS and stained with 0.1% crystal violet. The stained cells were imaged using the ImagePro 6.2 software. Cell counts were obtained from five random fields at 100× magnification. For the invasion assay, the membrane was coated with 30 mg/cm^2^ Matrigel (ECM gel, Sigma–Aldrich, St. Louis, MO, USA) to form a matrix barrier. The procedure followed for the invasion assay was the same as that for the migration assay except that the permeating time for the cells was 48 h.

### Terminal Deoxynucleotidyl Transferase dUTP Nick End Labeling (TUNEL) Assay

Apoptosis status of Huh7 cells was determined using a DeadEnd^TM^ Fluorometric TUNEL Assay Kit (Promega, Madison, WI) according to the manufacturers' protocol. In brief, Huh7 cells were treated with HGK (40 μM), sorafenib (5 μM), or both, for 48 h. The cells were then subjected to a TUNEL assay. The cells were then counted using a microscope (magnification, × 100). Cells in five different microscopic fields/dish were analyzed for each experiment.

### Cell-Cycle Analysis

Cells were trypsinized, washed twice, and incubated in PBS containing 0.12% Triton X-100, 0.12 mmol/L EDTA, and 100 mg/mL ribonuclease A. Propidium iodide (50 μg/mL) was then added to each sample for 20 min at 4°C. Cell cycle distribution was analyzed by flow cytometry (Beckman Coulter Epics Elite, Beckman, Inc.).

### *In vivo* Study

Six-week-old male BALB/c nude mice were purchased from the National Laboratory Animal Center (Taipei, Taiwan), maintained under specific pathogen-free conditions, and manipulated according to protocols approved by the Institutional Animal Care and Use Committee (IACUC) of Chang Gung Memorial Hospital (IACUC approval no.: 2018031301, approval date: 6/19/2018). A total of 5 × 10^6^ Huh7 cells were resuspended in 100 μl of saline with 50% Matrigel (BD Biosciences) and the suspensions were subcutaneously implanted into the left and right flank regions of the mice. All tumors were allowed to grow for 1 wk prior to the initiation of drug treatments. At the start of the second week, the mice with tumors were intraperitoneally injected 3 d/wk with 100 μl of HGK (1 mg/kg of body weight), sorafenib (15 mg/kg), or an equal volume of DMSO, which served as a control. Subcutaneous growth of the tumors was measured every 3 d and tumor volumes were calculated using the following equation: length × width^2^ × 0.5. Twenty-one days after drug administration, the mice were sacrificed and the tumors were subjected to immunohistochemical staining and analysis.

### Immunohistochemistry

The tumors of the mice were fixed in formalin and embedded in paraffin. Consecutive 2-μm-thick sections were cut from the paraffin-embedded tissue blocks and floated onto glass slides. The slide-mounted tissue sections were subjected to immunohistochemical staining as described previously (35).

### Chromatin Immunoprecipitation (ChIP)-qPCR Analysis

Chromatin immunoprecipitation assays were carried out using an Acetyl-Histone H3 Immunoprecipitation Assay Kit (Merck Millipore, Temecula, CA) according to manufacturer's instruction. Each of the purified DNAs (5 μl) were used as template for 60 cycles of PCR amplification using p21 promoter-specific primers (36) and TOOLS 2x SYBR qPCR Mix (BIOTOOLS CO., LTD., Taiwan).

### Statistical Analysis

All data were recorded as continuous variants and analyzed using Student's *t*-test. All statistical analyses were performed using SPSS 16.0 and Excel 2007 software. All statistical tests were two-sided and the *p*-values of significance were established at < 0.05 (^*^), < 0.01 (^**^), or < 0.001 (^***^), as indicated.

## Results

### HGK Inhibited the Expression of Class I HDAC in Yeast

**Figure 1:**
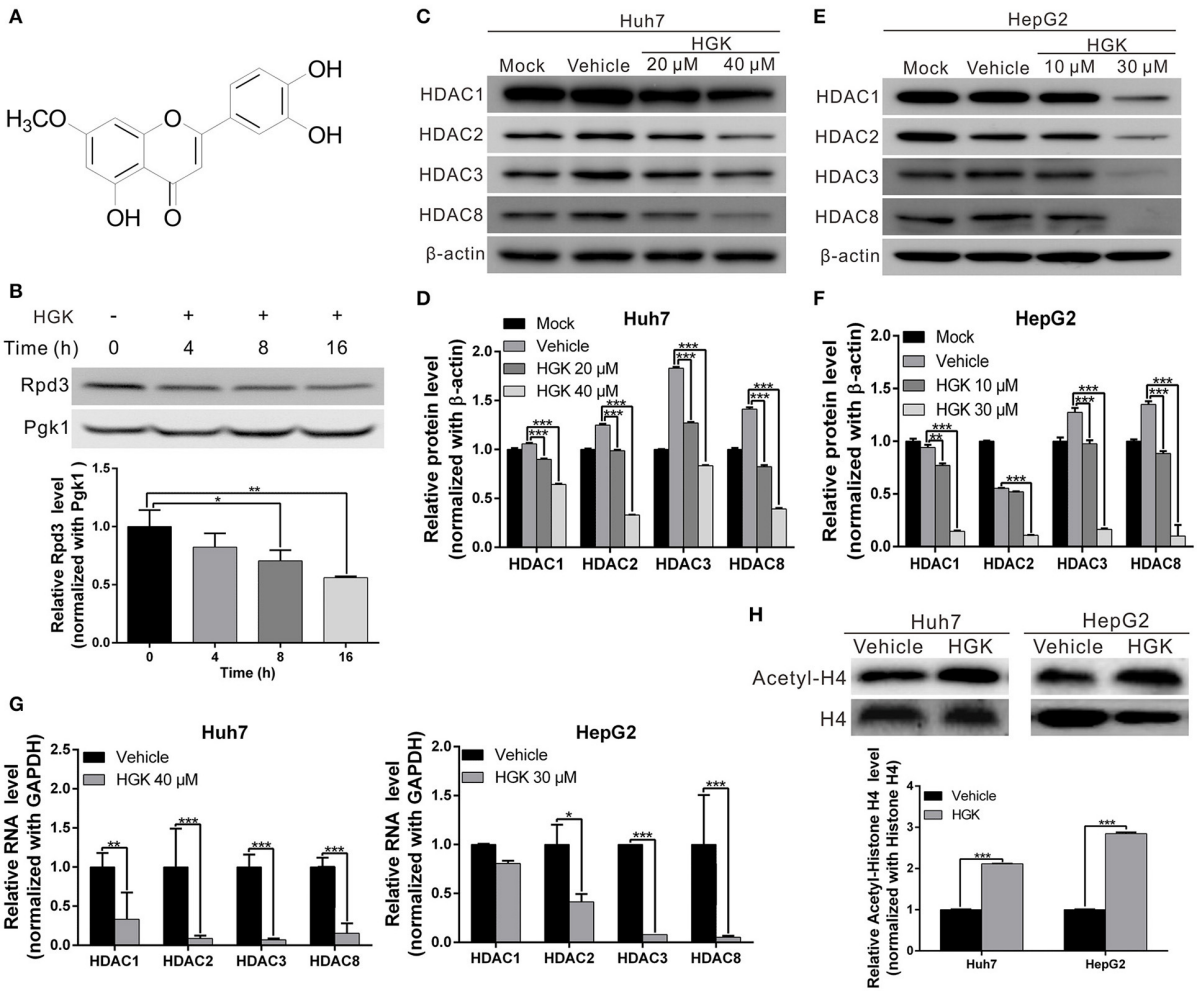
Hydroxygenkwanin (HGK) inhibited the expression of class I histone deacetylase (HDAC) in yeast and human liver cancer cell lines. **(A)** Chemical structure of HGK. **(B)** Western blot analysis revealed the effect on Rpd3 expression in yeast after the 48 h of 1 mM HGK treatment. Quantitative results are shown in lower panel. *p* < 0.05 (*), *p* < 0.01 (**), as assessed using the Student's *t*-test. **(C,E)** Huh7 and HepG2 cells were treated with HGK or vehicle for 48 h. The expression levels of HDAC 1, 2, 3, and 8 were determined using western blotting. Quantitative results are shown **(D,F)**. **(G)** RNA expression levels of HDAC 1, 2, 3, and 8 in Huh7 and HepG2 cells following treatment with vehicle (DMSO) or HGK analyzed by quantitative real-time RT-PCR. **(H)** The levels of acetylated histone H4 were determined by western blotting after 48 h of HGK treatment. Quantitative results are shown in the lower panel. *p* < 0.001 (***). All data are expressed as the mean ± standard deviation (SD) of three independent experiments.

Previous studies have shown that over-expression of class I HDAC is closely related to the development and progression of liver cancer. To identify natural compounds with inhibitory activity against class I HDAC, we used a yeast analysis platform previously established for drug screening (37). After treating the yeasts with different natural compounds, western blotting was performed to analyze the expression of reduced potassium dependency 3 (Rpd3) corresponding to human class I HDAC. We found that the amount of Rpd3 expressed in yeast treated with HGK was significantly lower compared to that in the vehicle control group (Figures 1A,B). The results showed that HGK may have inhibitory activity against class I HDAC in human cell lines.

### HGK Inhibited Class I HDAC Expression and Suppressed Proliferation, Migration, and Invasion Capacities of Liver Cancer Cells

To determine whether HGK inhibits the expression of class I HDAC in liver cancer cell lines, western blotting was used to analyze class I HDAC expression in HepG2 and Huh7 cells following treatment with different concentrations of HGK. The results demonstrated that expression levels of HDAC 1, 2, 3, and 8 were significantly decreased by HGK treatment in a dose-dependent manner (Figures 1C–G), suggesting that HGK was able to inhibit class I HDAC expression in liver cancer cells. In order to further understand the effect of HGK on the acetylation of histone proteins, we performed western blot analysis on the acetylation status of histone H4 in liver cancer cell lines treated with HGK. We found that, after treatment, the proportion of acetylated histone H4 was significantly increased compared with that of the vehicle group (Figure 1H), indicating that HGK promotes the acetylation of histone by inhibiting HDAC.

**Figure 2:**
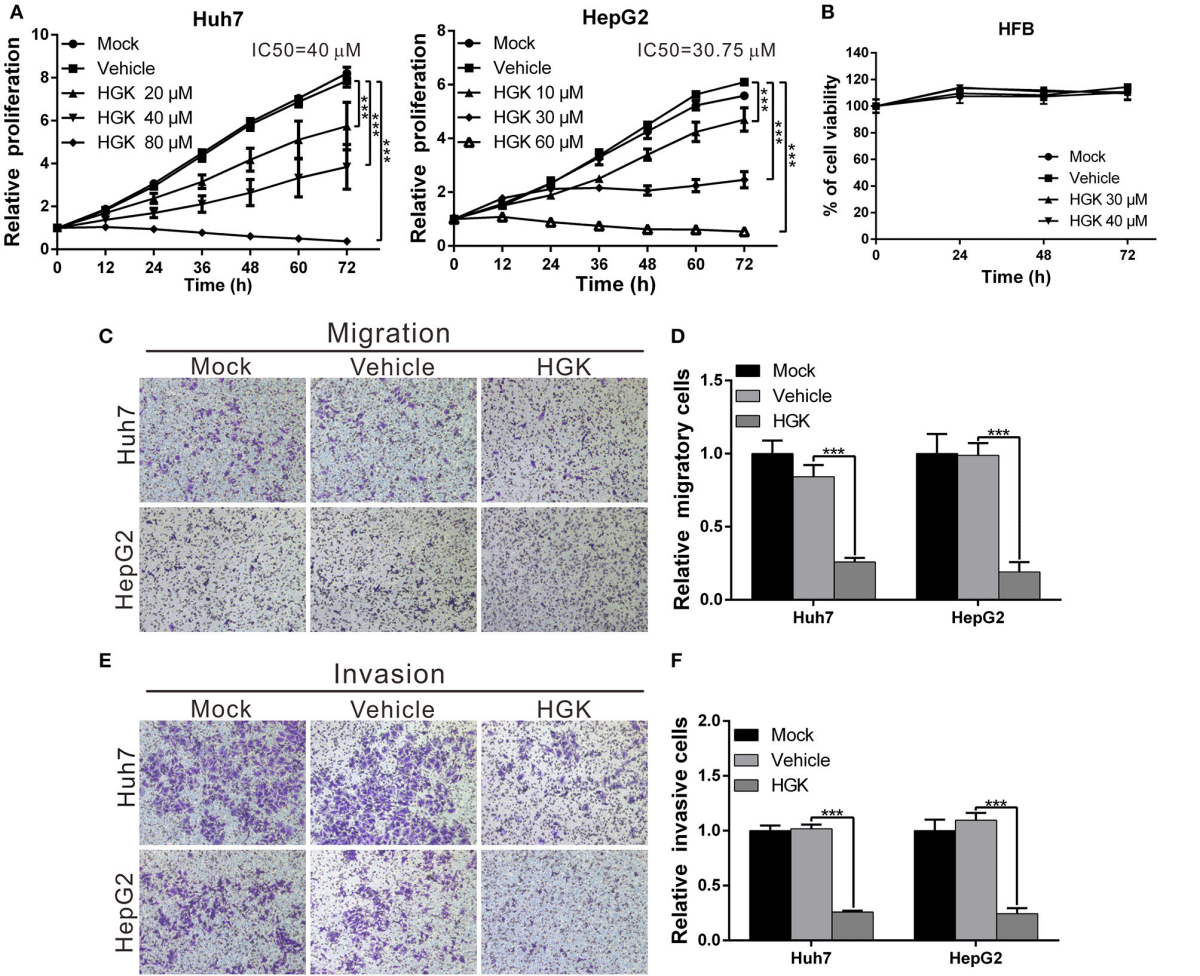
Hydroxygenkwanin (HGK) selectively inhibited the proliferation, migration, and invasion of hepatocellular carcinoma cells. **(A,B)** Huh 7, HepG2, and human skin fibroblast (HFB) cells were treated with different concentrations of HGK or vehicle (DMSO), and the cell proliferation status was analyzed using the xCELLigence Real-Time Cell Analyzer. **(C)** Cell migration capacities of Huh7 and HepG2 cells with/without HGK treatment (40 and 30 μM in Huh7 and HepG2 cells, respectively) were compared using transwell assays. The quantitative results are shown in **(D)**. **(E,F)** Invasion assays were performed using matrigel-coated polyethylene terephthalate membrane inserts. The results shown are the mean of three independent experiments. Significant differences vs. the control groups, *p* < 0.001 (***).

To elucidate the effect of HGK on the physiology of liver cancer cells, we performed cell functional analysis to detect changes in proliferation, migration, and invasion of liver cancer cell lines Huh7 and HepG2. The results revealed that proliferation, migration, and invasion abilities of the two cell lines were significantly lower in HGK-treated cells compared to those of control cells. Additionally, increased HGK concentrations resulted in greater inhibition (Figures 2A–F), showing that HGK may exert its anticancer activity by inhibiting class I HDAC. The half-maximal inhibitory concentration (IC_50_) of HGK toward Huh7 and HepG2 cells was calculated using GraphPad Prism software and was about 40 and 30 μM, respectively. However, no inhibitory effect was observed on the growth of human skin fibroblast cell line HFB at the above concentrations (Figure 2B). This indicated that HGK selectively inhibits the growth of liver cancer cells without significant toxicity to normal cells.

Moreover, we compared the efficacy of HGK against liver cancer with the clinically used HDAC inhibitor SAHA and its toxicity to normal cells. The half-maximal inhibitory concentration (IC_50_) of SAHA toward Huh7 and HepG2 cells was about 3 μM. We found that the physiological toxicity of HGK to human skin fibroblast (HFB) cell line was significantly lower than that of SAHA at the half maximal inhibitory concentration (Figure S1D). In addition, after 30 h of treatment, both HGK and SAHA significantly inhibited the growth of the liver cancer cell lines. However, the inhibitory effect of SAHA was better than that of HGK, as SAHA obviously triggered apoptosis (data not shown). Flow cytometry results also confirmed that the tumor suppressor mechanism of HGK mainly caused cell cycle arrest, while SAHA caused apoptosis (Figures S1A–C).

### HGK Effects on p21, p53, and p65 Gene

**Figure 3:**
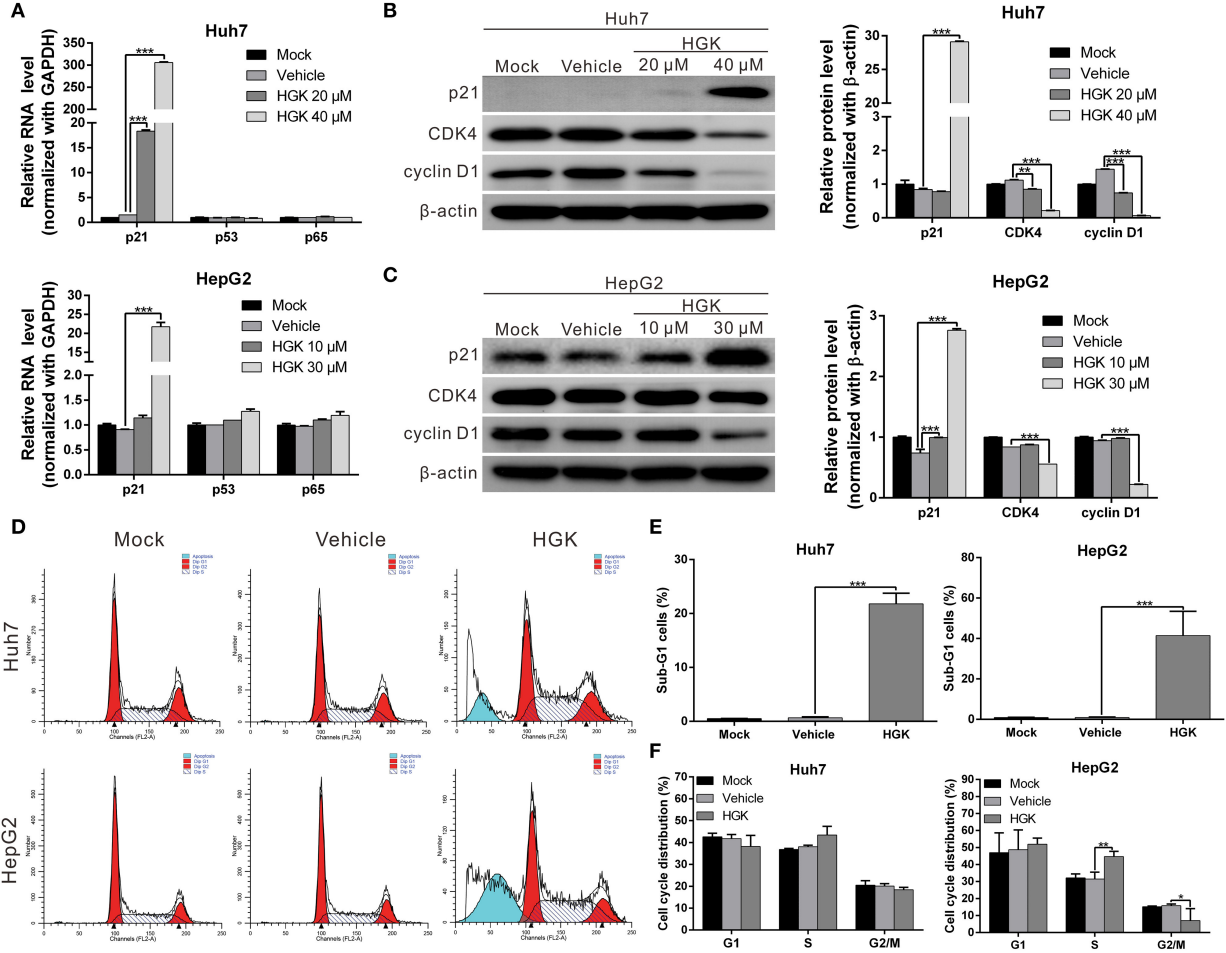
Hydroxygenkwanin (HGK) induced the expression of tumor suppressor gene p21. **(A)** Huh7 and HepG2 cells were treated with HGK or vehicle for 48 h and the RNA levels of p21, p53, and p65 were analyzed using quantitative real-time RT-PCR. **(B,C)** Expression levels of p21 and downstream proteins in Huh7 and HepG2 cells following treatment with vehicle (DMSO) or HGK analyzed by western blotting (left panel). β-actin served as an internal control. Quantitative results are shown in right panel. *p* < 0.01 (**), *p* < 0.001 (***). **(D–F)** Effect of HGK on the cell cycle progression in Huh7 and HepG2 cells. Cells were treated with vehicle or HGK for 48 h. The cell cycle distribution was analyzed by flow cytometry. The results shown are the mean of three independent experiments. *p* < 0.05 (*), *p* < 0.01 (**), *p* < 0.001 (***).

**Figure 4:**
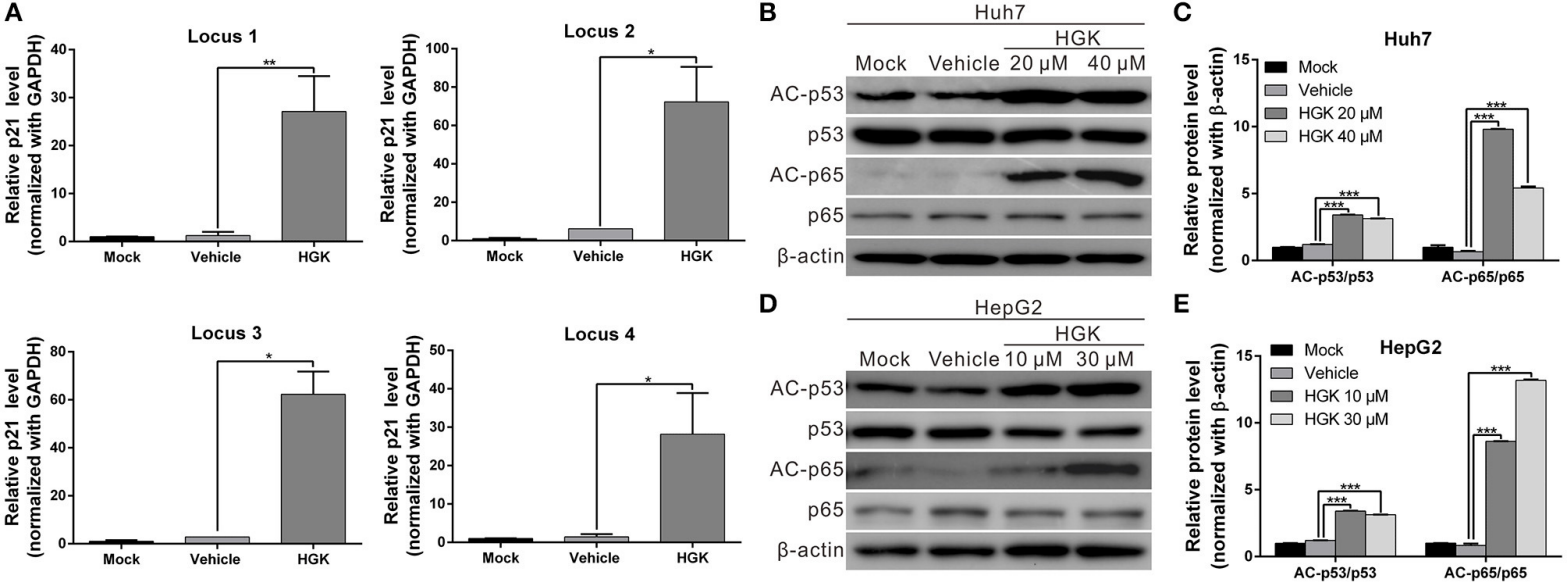
Hydroxygenkwanin (HGK) induced histone acetylation of p21 promoter and promoted the acetylation of p53 and p65 proteins. **(A)** Acetylation of histone H3 associated with the p21 promoter was increased in HGK treated cells. HepG2 cells were treated with HGK or vehicle for 48 h and subjected to ChIP-qPCR analysis using acetyl-histone H3 antibody. Precipitated genomic DNA was amplified for the four proximal promoters of the p21 locus by real-time PCR. **(B,D)** Huh7 and HepG2 cells were treated with HGK for 48 h and the expression and acetylation levels of p53 and p65 proteins were analyzed by western blotting. β-actin served as an internal control. Quantitative results are shown **(C,E)**. *p* < 0.05 (*), *p* < 0.01 (**), *p* < 0.001 (***). All data are expressed as the mean ± standard deviation (SD) of three independent experiments.

HDAC regulates the de-acetylation of histone and non-histone proteins, affecting gene expression or protein activation. Previous studies have shown that class I HDAC inhibits the expression of the tumor suppressor gene p21 and alters the acetylation and activation of p53 and p65 proteins, which in turn promote carcinogenesis (38–41). To determine the effect of HGK on tumor suppressor molecules, real-time RT-PCR and western blotting were used to analyze the expression of p21 and acetylation of p53 and p65 in liver cancer cell lines treated with HGK (Figures 3A–C). The results showed that compared with that in the vehicle control group, expression levels of p21 mRNA and protein significantly increased in Huh7 and HepG2 cells treated with HGK, which inhibited the expression of cell cycle regulatory proteins CDK4 and cyclin D1, thereby inducing S-phase cell cycle arrest and promoting apoptosis (Figures 3D–F). To further confirm that HGK increased expression from the p21 gene region by modulating acetylation of histones, we performed a chromatin immunoprecipitation (ChIP) assay. The assay demonstrated that the degree of histone H3 acetylation in four distinct p21 promoter regions were significantly increased following treatment with HGK (Figure 4A). This suggested that HGK enhanced p21 expression by promoting the acetylation of histone H3 in the p21 promoter region. Moreover, the degree of p53 and p65 protein acetylation in the two cell lines was significantly increased following HGK treatment (Figures 4B–E), showing that HGK altered the activation of tumor suppressor proteins, such as p53 and p65.

### Synergistic Effect of HGK and Sorafenib

**Figure 5:**
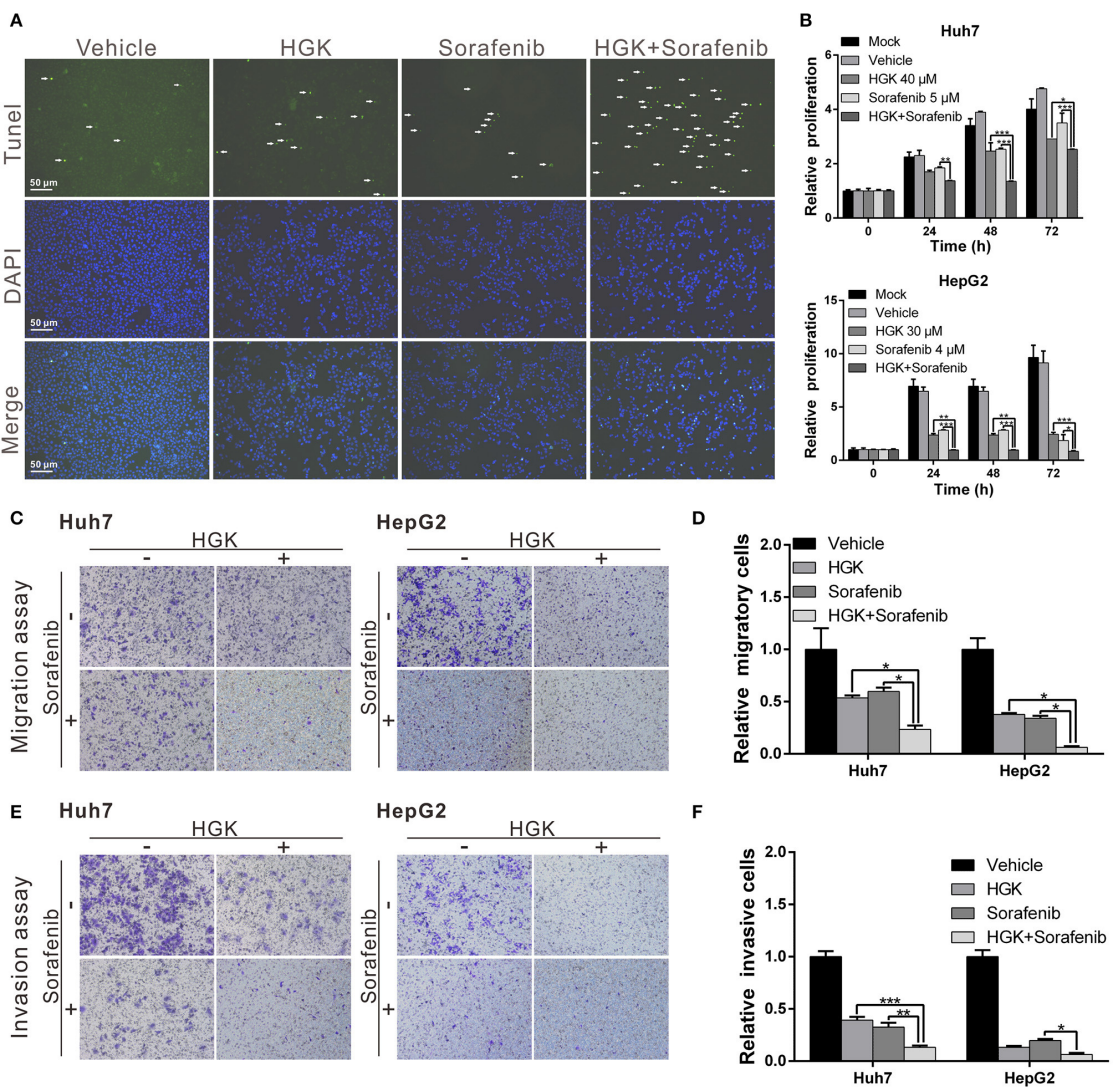
Hydroxygenkwanin (HGK) and sorafenib had synergistic effects against liver cancer cells. **(A)** Huh7 cells were treated with 40 μM HGK and 5 μM sorafenib individually or in combination and cell apoptosis was determined using a terminal deoxynucleotidyl transferase dUTP nick end labeling (TUNEL) assay. Green punctate staining represents TUNEL-positive cells. Apoptotic cells were identified as DAPI and TUNEL double-stained cells. Magnification: 100×. Effects of HGK (40 and 30 μM in Huh7 and HepG2 cells, respectively) combined with sorafenib (5 μM in Huh7 and 4 μM in HepG2 cells) on cell proliferation **(B)**, migration **(C,D)**, and invasion **(E,F)**. *p* < 0.05 (*), *p* < 0.01 (**), *p* < 0.001 (***). All experiments were performed in triplicate.

Previous studies have shown that HDAC inhibitors have synergistic effects with sorafenib that enhance cancer suppression efficacy. To determine whether HGK and sorafenib had synergistic effects, liver cancer cell lines were treated with HGK and sorafenib, separately and combined, and the cells were analyzed for apoptosis. The results demonstrated that treatment with HGK or sorafenib alone slightly induced cell apoptosis. However, when both compounds were administered simultaneously, the cytotoxic effects on the cell lines were significantly greater compared with that observed in cells that received single-drug treatments (Figure 5A). Moreover, the combination of the two drugs inhibited cancer cell proliferation, migration, and invasion by more than 1-fold compared to the inhibition by either HGK or sorafenib alone (Figures 5B–F). These results confirmed that the combined use of HGK and sorafenib had synergistic effects against liver cancer cells.

### Combination of HGK and Sorafenib Enhanced Efficacy Against Liver Cancer *in vivo*

**Figure 6:**
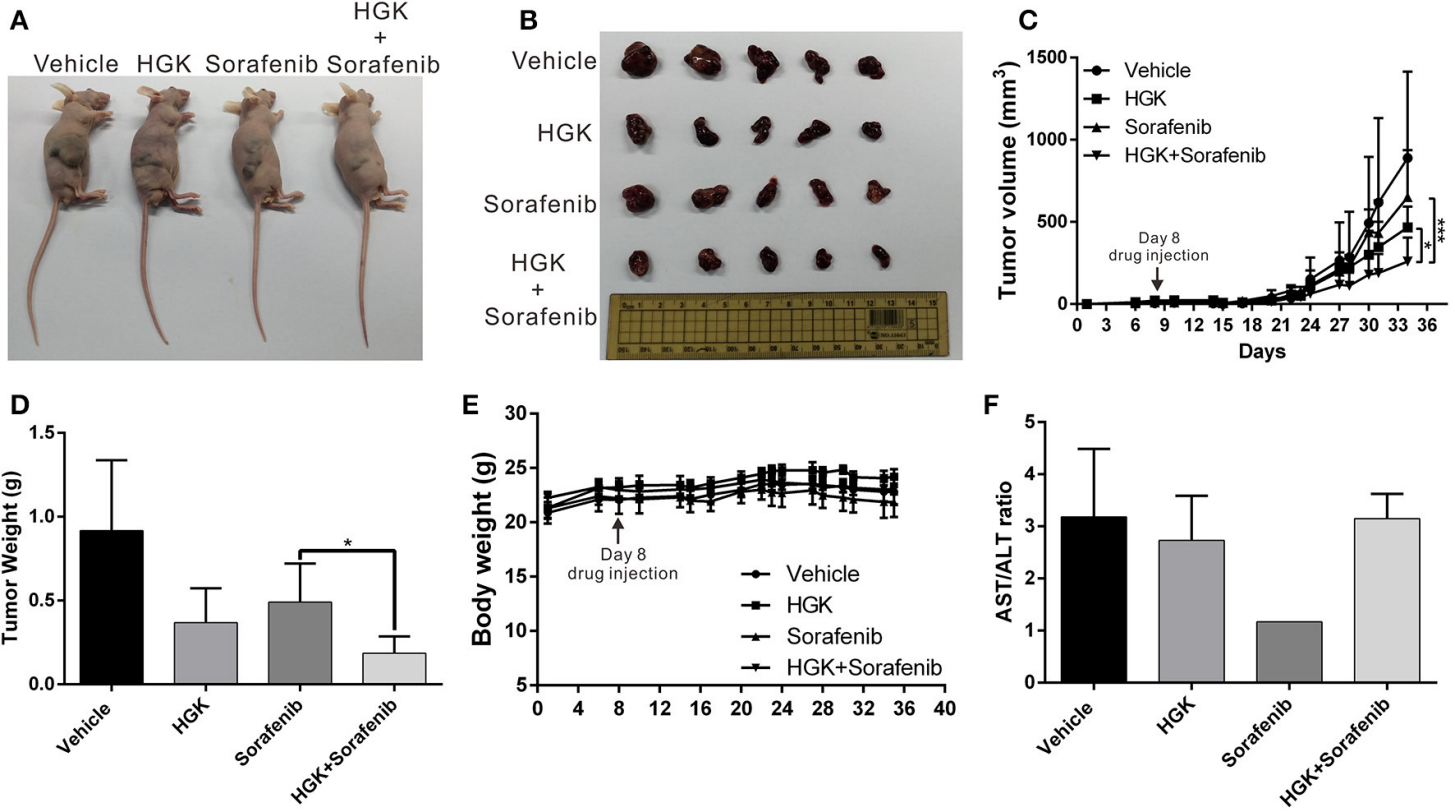
Combination of hydroxygenkwanin (HGK) and sorafenib enhanced *in vivo* anticancer efficacy. **(A,B)** A total of 5 × 106 Huh7 cells were inoculated into nude mice (*n* = 5 per group). The mice with tumors were intraperitoneally injected three times per wk with 100 μl of HGK (1 mg/kg of body weight), sorafenib (15 mg/kg), or an equal volume of dimethyl sulfoxide (DMSO), which served as a control. Representative images show the tumor xenografts at 4 wk post-implantation. **(C)** Tumor volumes were recorded every 3 d after injection as follows: length × width^2^ × 0.5. The error bars indicate the S.D. *p* < 0.05 (*), *p* < 0.001 (***). Tumor weights **(D)** and body weights **(E)** were calculated every 3 d after injection. **(F)** Serological test results of the four groups of mice.

To verify that HGK had anticancer effects *in vivo* and to confirm the regulatory mechanisms involved, a mouse xenograft model was used. Huh7 cells were injected into the back of mice and HGK and sorafenib were periodically administered by intraperitoneal injection, either separately or in combination. The results showed that tumor growth in mice treated with HGK, sorafenib, or both was significantly inhibited compared with that in the vehicle dimethyl sulfoxide (DMSO)-treated group. Moreover, the inhibitory effects of the combination treatment on the tumors were significantly greater compared to that of single-drug treatment (Figures 6A–D), showing a synergistic efficacy for HGK and sorafenib. Additionally, there was no significant difference in body weight between the mice given HGK alone and the vehicle group (Figure 6E), nor were there any significant abnormalities in the serological test results for the two groups of mice (Figure 6F). This indicated that HGK failed to demonstrate any noteworthy physiological toxicity.

**Figure 7:**
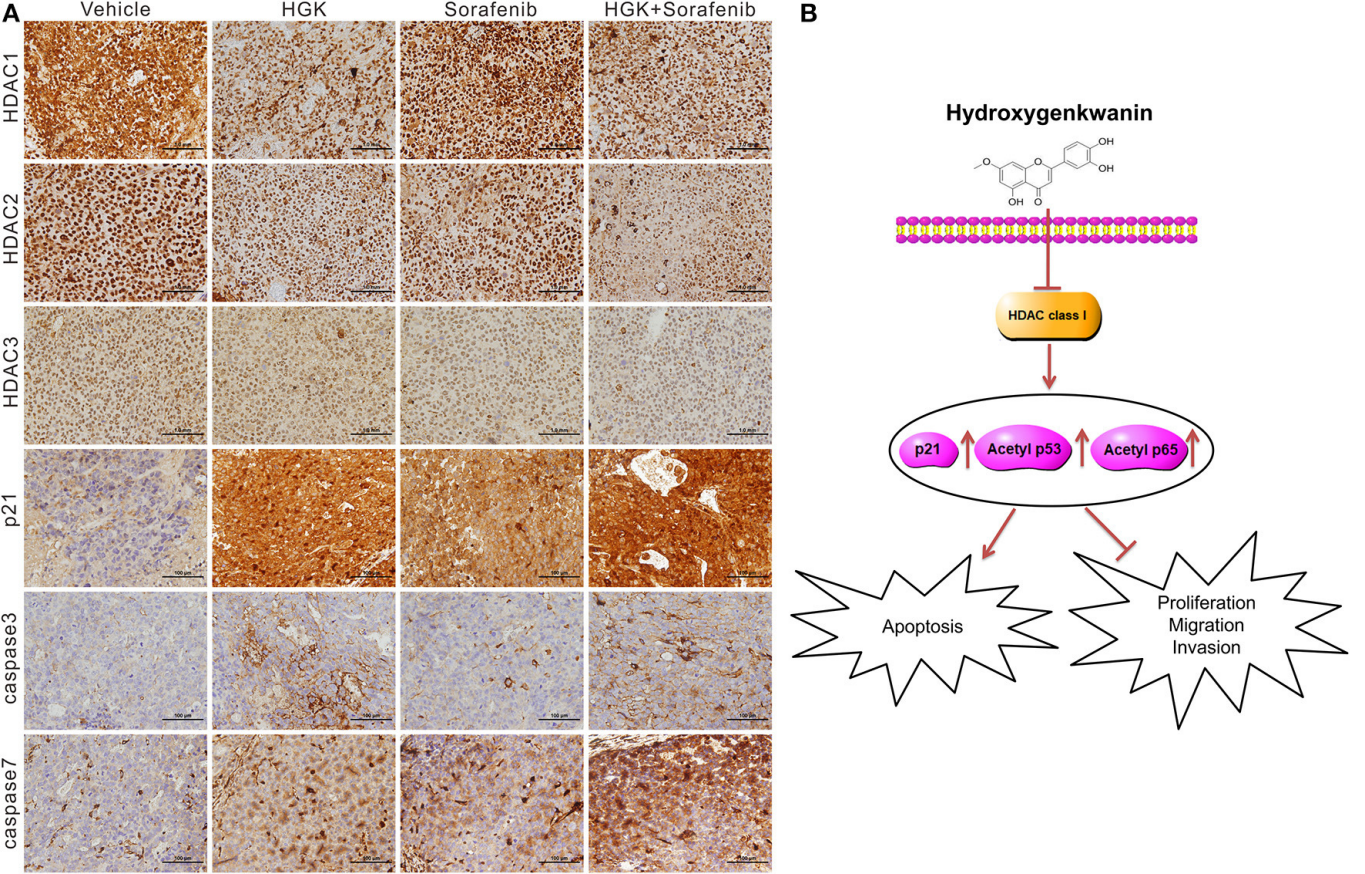
Hydroxygenkwanin (HGK) repressed tumor growth by inhibiting class I histone deacetylase (HDAC). **(A)** Immunohistochemical staining representing the effect of HGK on class I HDACs, p21, caspase 3, and caspase 7 expression in mice xenograft tumors. Magnification: 400×. **(B)** Schematic representation summarizing the anti-hepatocellular carcinoma mechanism of HGK.

In addition, expression of class I HDAC genes was analyzed using immunohistochemical staining of mouse tumor tissues. Interestingly, the results were consistent with those from the *in vitro* experiments. Expression levels of class I HDAC were significantly decreased in mouse tumors treated with HGK (Figure 7A). These results confirmed that HGK induced expression of a tumor suppressor genes by inhibiting class I HDAC, thereby promoting apoptosis in liver cancer cells and inhibiting tumor growth (Figure 7B).

## Discussion

Abnormal regulation of epigenetics is closely related to tumorigenesis, which is partly regulated by HDAC. Previous studies have demonstrated that class I HDACs are highly expressed in liver cancer tissues and closely associated with tumor development and progression, which makes them important targets for liver cancer treatment (42–44). In the current study, we determined that the natural compound HGK possessed class I HDAC inhibitory activity and low physiological toxicity. We also confirmed that HGK induced expression or activation of p21, p53, p65, and other tumor suppressor genes to inhibit the growth of liver cancer tumors. Importantly, we determined that HGK exhibited a synergistic effect with sorafenib in enhancing tumor-suppressing effects. To our knowledge, this is the first study to identify HGK as a class I HDAC inhibitor and to demonstrate its therapeutic potential for liver cancer treatment.

HDAC regulates gene expression and protein activation by regulating the acetylation of histone and non-histone proteins; however, the regulatory mechanism of cancer physiology is extremely diverse and complex and the carcinogenic and tumor-suppressing pathways regulated in various cancers are different. In the current study, we limited our analyses to tumor suppressor genes such as p21, p53, and p65, which explains only part of the anticancer mechanism of HGK. We believe that there are multiple key factors involved; however, this needs to be clarified with further experiments.

In the present study, we found that HGK promoted histone acetylation in the p21 promoter region, which in turn upregulated p21 expression. p21 is a cyclin-dependent kinase inhibitor (CKI) that acts on cyclin-dependent kinases (CDKs), consequently inhibiting cell cycle progression (45). The p21 gene is also one of the genes regulated by p53. Previous studies have shown that acetylation of the p53 protein enhances its stability allowing it to bind to the target gene promoter, thereby upregulating the expression of tumor suppressor genes, such as p21 and BAX (46, 47). The results of the present study indicate that HGK can promote the acetylation of p53 at lysine 382 (K382), suggesting that HGK can also indirectly promote p21 expression through the regulation of p53 acetylation (48). Furthermore, previous research has determined that acetylation at different sites on the p65 protein produces different effects on p65 activity (49). For example, acetylation of p65 at K310 enhances the ability of p65 to bind to the target gene promoter, which in turn regulates the expression of tumor suppressor genes, such as miR21 and DR5, and activates the downstream pathways. Therefore, the acetylation of p65 at K310 is also regarded as an indicator of the anticancer activity of p65. In the present study, we found that p65 acetylation at K310 was significantly increased in HGK-treated cells, showing that HGK can achieve anticancer effects by activating p65 and upregulating the expression of its downstream tumor suppressor genes.

Currently, histone deacetylase inhibitors (HDACi) are widely used in cancer treatment and have achieved good therapeutic effects in treating various cancers. Suberoylanilide hydroxamic acid (SAHA) and romidepsin, two widely studied HDACi, have been approved by the US FDA for treatment of T-cell lymphomas (50). Many studies have shown that these HDACi can also inhibit the progression of cancers such as breast cancer, lung cancer, and prostate cancer (51–54). Furthermore, many clinical trials have demonstrated that the concomitant use of HDACi and other anticancer drugs provides synergistic therapeutic effects (55–57). For instance, the concomitant use of SAHA and bortezomib can synergistically induce ROS-driven caspase-dependent apoptosis of nasopharyngeal carcinoma (58); the concomitant use of romidepsin with cisplatin and nivolumab can enhance the therapeutic effects of the individual medications on triple-negative breast cancer; and a combination therapy using pralatrexate and romidepsin enhances the therapeutic effects on relapsed and refractory T-cell lymphoma [56]. Studies on liver cancer have also indicated a similar synergistic killing effect on liver cancer cell lines when using the HDACi valproic acid (VPA) along with aspirin (59). In the present study, we found that the concomitant use of HGK and sorafenib significantly enhanced their inhibitory effects on the growth and metastasis of malignant liver tumors. Thus, the different mechanisms by which HGK and sorafenib achieve their anticancer effects result in synergism during concomitant use. This result also suggests that HGK can potentially be used as an adjuvant agent in clinical treatment.

SAHA is an HDAC inhibitor that is currently approved by the FDA for the treatment of T-cell lymphoma. It can specifically bind to the zinc-containing catalytic domains of class I, II, and VI histone deacetylases (HDACs), thereby suppressing their enzymatic activities. However, HGK inhibits the growth of liver cancer cells by inhibiting the expression of class I HDAC, which is different from the mechanism of action of SAHA. In addition, in our recent study, we found that HGK can also inhibit the expression of FOXM1, thereby inhibiting EMT progression by regulating miR320a expression (60). The anti-HCC effect of HGK is known to involve two or more regulatory mechanisms. In this study, we aimed to show that HGK can inhibit the progression of liver cancer by inhibiting the expression of class I HDAC. Although its efficacy and specificity are not as good as SAHA, the results of our study indicate that HGK has lower physiological toxicity to normal cells and can be potentially used as a therapeutic adjuvant in the treatment of liver cancer.

Class I HDAC is highly expressed in most tumor tissues, including liver cancer, breast cancer, lung cancer, and colorectal cancer, and is one of the main targets for cancer treatment (61–64). Therefore, we focused on the regulation of class I HDAC by HGK. However, in yeast, HGK also inhibited the expression of Sir2, suggesting that it may also have class III HDAC inhibitory activity in human cells (data not shown). Previous studies have shown that class III HDAC is closely related to tumorigenesis and prognosis in leukemia, glioblastoma, prostate cancer, colorectal cancer, and skin cancer (65–69). Therefore, we plan to continue exploring whether HGK inhibits human class III HDAC and to test its inhibitory effect on multiple cancer cell types in effort to assess whether HGK might be used to treat other types of cancer. In addition, further studies are needed to understand how HGK upregulates HDACs expression.

Although high HDAC expression levels are closely related to tumor progression and patient outcome in most cancers, HDAC deactivation due to gene mutations has been observed in some cancers. For example, HDAC1 somatic mutations have been found in 8.3% of dedifferentiated liposarcoma and HDAC4 homozygous deletions are found in 4% of melanoma (70, 71). These mutations result in deactivation of HDAC and slows tumor growth. However, since mutations increase resistance to HDAC inhibitors, it is speculated that this is one reason why many cancers, including liver cancer, are not sensitive to HDAC inhibitors. Therefore, the combination of different drugs in cancer treatment is crucial for improving efficacy.

## Conclusions

While HDAC inhibitors have been widely used in treating various cancers, their side effects cause bottlenecks in treatment. In the current study, we identified HGK, a natural compound with class I HDAC inhibitory activity, as an anticancer compound that acted synergistically with sorafenib. Importantly, since HGK is not physiologically toxic, it is suitable as a therapeutic adjuvant in combination with other anticancer drugs to enhance their therapeutic effects.

## Data Availability Statement

All datasets generated for this study are included in the article/Supplementary Material.

## Ethics Statement

The animal study was reviewed and approved by Institutional Animal Care and Use Committee (IACUC) of Chang Gung Memorial Hospital, Taiwan (IACUC approval no.: 2018031301, approval date: 6/19/2018).

## Author Contributions

Conceptualization: T-HW and C-CC. Data curation: C-YC and T-HW. Formal analysis: C-TY, W-YC, and Y-LL. Investigation and project administration: T-HW, C-CC, and C-YC. Methodology: CH and C-TY. Resources: Y-LL and CH. Supervision: T-HW. Validation: S-HU. Visualization: S-HU, W-YC, and CH. Writing of the original manuscript draft and manuscript review and editing: T-HW and C-YC. All authors contributed to manuscript revision and have read and approved the submitted version.

### Conflict of Interest

The authors declare that the research was conducted in the absence of any commercial or financial relationships that could be construed as a potential conflict of interest.

## Supplementary Material

Figure S1 Comparison of antiproliferative effect and physiological toxicity of HGK and SAHA. **(A,B)** Effect of HGK and SAHA on the cell cycle progression in Huh7 and HepG2 cells. Cells were treated with or without stated concentrations of HGK or SAHA for 30 h. The cell cycle distribution was analyzed by flow cytometry. The quantitative results were shown in **(C)**. **(D)** Human skin fibroblast (HFB) cells were treated with stated concentrations of HGK, SAHA, or vehicle (DMSO), and the cell viability was analyzed using the xCELLigence Real-Time Cell Analyzer. The results shown are the mean of three independent experiments. Significant differences versus the control group (vehicle), ^***^*p* < 0.001.
