# Supplementary figures and images for: Hydroxygenkwanin Inhibits Class I HDAC Expression and Synergistically Enhances the Antitumor Activity of Sorafenib in Liver Cancer Cells
Source: Front Oncol. 2020 Feb 25;10:216. doi: 10.3389/fonc.2020.00216 (PMC7052045; doi:10.3389/fonc.2020.00216)

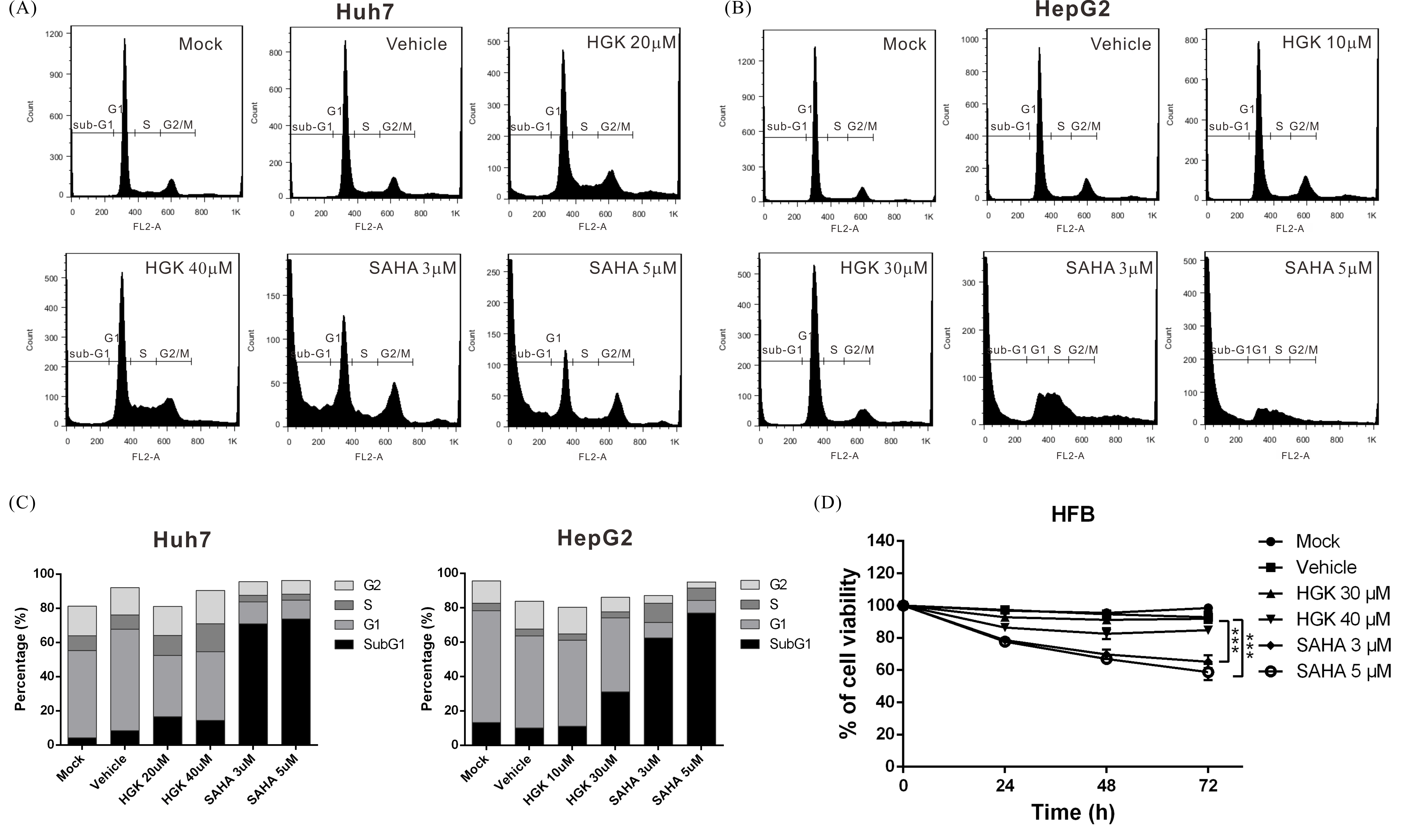

Supplement: Figure S1 — Comparison of antiproliferative effect and physiological toxicity of HGK and SAHA. (A,B) Effect of HGK and SAHA on the cell cycle progression in Huh7 and HepG2 cells. Cells were treated with or without stated concentrations of HGK or SAHA for 30 h. The cell cycle distribution was analyzed by flow cytometry. The quantitative results were shown in (C). (D) Human skin fibroblast (HFB) cells were treated with stated concentrations of HGK, SAHA, or vehicle (DMSO), and the cell viability was analyzed using the xCELLigence Real-Time Cell Analyzer. The results shown are the mean of three independent experiments. Significant differences versus the control group (vehicle), ***p < 0.001. [file Image_1.tif]
